# Mechanistic Insights Into MicroRNA-Induced Neuronal Reprogramming of Human Adult Fibroblasts

**DOI:** 10.3389/fnins.2018.00522

**Published:** 2018-08-02

**Authors:** Ya-Lin Lu, Andrew S. Yoo

**Affiliations:** ^1^Department of Developmental Biology, School of Medicine, Washington University in St. Louis, St. Louis, MO, United States; ^2^Program in Developmental, Regenerative and Stem Cell Biology, School of Medicine, Washington University in St. Louis, St. Louis, MO, United States

**Keywords:** microRNA, chromatin, neuronal conversion, reprogramming, neurogenesis, disease modeling, human neurons

## Abstract

The use of transcriptional factors as cell fate regulators are often the primary focus in the direct reprogramming of somatic cells into neurons. However, in human adult fibroblasts, deriving functionally mature neurons with high efficiency requires additional neurogenic factors such as microRNAs (miRNAs) to evoke a neuronal state permissive to transcription factors to exert their reprogramming activities. As such, increasing evidence suggests brain-enriched miRNAs, miR-9/9^∗^ and miR-124, as potent neurogenic molecules through simultaneously targeting of anti-neurogenic effectors while allowing additional transcription factors to generate specific subtypes of human neurons. In this review, we will focus on methods that utilize neuronal miRNAs and provide mechanistic insights by which neuronal miRNAs, in synergism with brain-region specific transcription factors, drive the conversion of human fibroblasts into clinically relevant subtypes of neurons. Furthermore, we will provide insights into the age signature of directly converted neurons and how the converted human neurons can be utilized to model late-onset neurodegenerative disorders.

## Introduction

Overcoming epigenetic barriers through direct cellular reprogramming has allowed scientists to rapidly acquire cell types of interest for regenerative therapies and disease modeling. Direct conversion of mouse fibroblasts into functional neurons have been demonstrated through the use of transcription factors (Vierbuchen et al., 2010; Son et al., 2011; Chanda et al., 2014; Blanchard et al., 2015). Empirically, however, obtaining mature human neurons from adult human fibroblasts with transcription factors have been challenging (Caiazzo et al., 2011). To enhance reprogramming efficiency and to promote neuronal maturation, small chemical molecules (Ladewig et al., 2012; Liu et al., 2013; Pfisterer et al., 2016; Smith et al., 2016) and RNA molecules, miRNAs (Ambasudhan et al., 2011; Yoo et al., 2011; Victor et al., 2014; Abernathy et al., 2017), have been used in conjunction with transcription factors to robustly generate functional neurons from human fibroblasts. The mechanism(s) by which miR-9/9^∗^ and miR-124 collectively drive robust neuronal fate conversion remains an ongoing investigation, but through examining transcriptome and epigenetic changes at the genome-wide level, we attempt to elucidate how miRNAs promote the neuronal identity during direct conversion of human fibroblasts to neurons.

## miRNAs as Potent Cell Fate Regulators

Traditionally, transcription factors, in particular, pioneer transcription factors, have been viewed as regulators and determinants of cell fate. With domains that can interact directly with chromatin and/or other modifier proteins, transcription factors have been widely used for cellular reprogramming (Iwafuchi-Doi and Zaret, 2014, 2016), including the generation of induced pluripotent stem cells (iPSC) from somatic cells (Takahashi and Yamanaka, 2006). Increasing studies across different cellular contexts have revealed that miRNAs are also potent cell fate regulators as miRNAs not only target large repertoire of genes in genetic networks but also epigenetic regulators necessary for the remodeling of the chromatin (Yoo et al., 2009; Ivey and Srivastava, 2010; Gruber and Zavolan, 2013; Rajman and Schratt, 2017). Subsequently, miRNAs have been used to generate iPSCs (Anokye-Danso et al., 2011), cardiomyocytes (Jayawardena et al., 2012), and neurons (Yoo et al., 2011) from fibroblasts.

## miR-9/9^∗^ and miR-124 are Neurogenic Molecules

The acquisition of neuronal fate requires the downregulation of the neuron-restrictive silencer factor (NRSF) or repressor element-1 silencing transcription factor (REST) that represses neuronal genes in non-neuronal cells, including the neuron-specific miRNAs, miR-9/9^∗^, and miR-124 (miR-9/9^∗^-124) (Lagos-Quintana et al., 2002; Lim et al., 2005; Conaco et al., 2006; Deo et al., 2006). Both miR-9/9^∗^ and miR-124 are highly abundant in neuronal tissues (Lagos-Quintana et al., 2002; Lim et al., 2005; He et al., 2012), and are essential for neuronal differentiation (Cheng et al., 2009; Dajas-Bailador et al., 2012; Xue Q. et al., 2016) and the maintenance of neuronal identity through the repression of anti-neural genes including cofactors of the REST complex, RCOR1 and SCP1 (Visvanathan et al., 2007; Packer et al., 2008). As overexpression of miR-9/9^∗^ (Leucht et al., 2008; Zhao et al., 2009) and/or miR-124 (Krichevsky et al., 2006; Cheng et al., 2009; Akerblom et al., 2012) in stem cells or neural progenitors resulted in the precocious acquisition of neuronal fate, demonstrating the function of miRNAs in the activation of neuronal program (Lim et al., 2005). Ectopic expression of miR-9/9^∗^-124 was also shown to drive the direct conversion of primary human dermal fibroblasts into functional neurons (Yoo et al., 2011). Therefore, knockdown of REST is sufficient to promote neuronal identity in part due to miR-9/9^∗^-124-dependent mechanisms (Drouin-Ouellet et al., 2017). Furthermore, miR-9/9^∗^-124 orchestrates the reduction of REST protein stability during neuronal reprogramming to promote chromatin accessibility of neuronal loci (Lee et al., 2018) and induction of neuronal genes (Abernathy et al., 2017; Drouin-Ouellet et al., 2017; Lee et al., 2018). Interestingly, miR-124 alone has also been used in neuronal conversion with the help of transcription factors (Ambasudhan et al., 2011; Jiang et al., 2015). Here, we review current understanding of the properties of miR-9/9^∗^ and miR-124 in both developmental and cellular reprogramming contexts highlighting their synergistic roles in coordinating the molecular switching of several critical non-neuronal to neuronal components during mammalian neurogenesis. We will mainly focus on the molecular switches critical in epigenetic regulation such as chromatin remodeling and DNA methylation, and transcriptome dynamics such as alternative splicing underlying the adoption of the neuronal identity. Our discussion will also include molecular pathways that occur during *in vivo* neurogenesis and are also recapitulated in the miRNA-directed reprogramming of human fibroblasts into neurons for the successful overcoming of cell fate barriers.

## miRNAs Orchestrate the Composition of BAF Chromatin Remodeling Complexes

Spatial and temporal reciprocity of homologous gene or isoform expression during neurogenesis is a recurring theme. Previous studies have indicated that the neurogenic and reprogramming activity of miR-9/9^∗^ and miR-124 may be in part through the direct targeting of subunits of the ATP-dependent BRG/BRM associated factor (BAF) chromatin remodeling complexes (Yoo et al., 2009; Staahl and Crabtree, 2013; Staahl et al., 2013). Mammalian BAF complexes are large multi-subunit complexes combinatorically assembled in a cell type-dependent manner. The combinatorial assembly of different homologs and splice variants of BAF subunit families confers functional specificity as each subunit contains functional domains that recognized DNA and/or modified histones (Wu et al., 2009; Zheng et al., 2012). For example, embryonic stem cell (ESC) BAF (esBAF) is characterized by BAF53a and a homodimer of BAF155, as opposed to a heterodimer of BAF155 and BAF170 in differentiated cells (Wang et al., 1996a,b; Ho et al., 2009b). The esBAF complex is involved in maintaining pluripotency by establishing an ESC-specific chromatin state permissive for transcription factors and signaling molecules to access ESC-associated genes (Ho et al., 2009a; Kidder et al., 2009). Although BAF complexes are traditionally known to antagonize the function of polycomb repressive complexes (PRC) to promote chromatin accessibility for gene activation (Kennison, 1995; Ho et al., 2011), studies have also suggested that BAF complexes can synergize with PRC for gene regulation (Ho et al., 2011).

**FIGURE 1:**
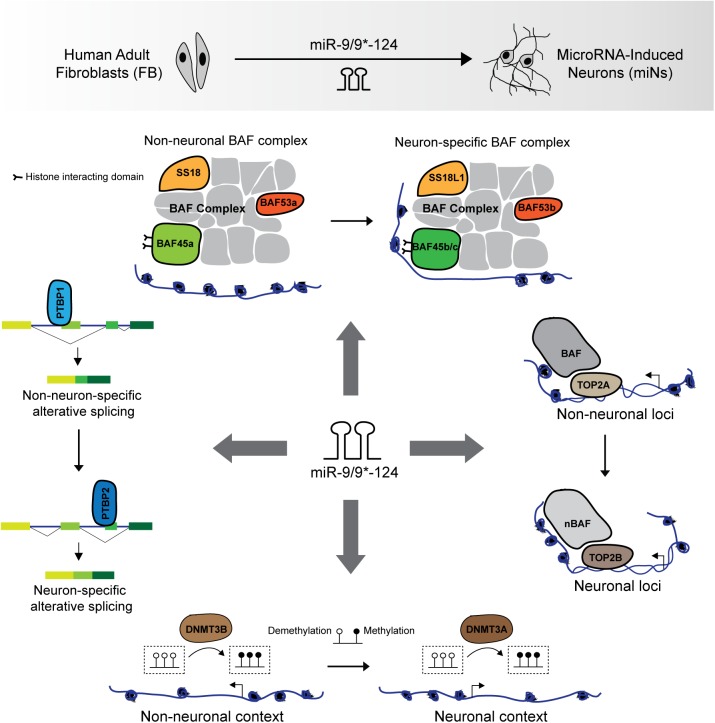
miR-9/9^∗^-124-mediated neuronal reprogramming of human adult fibroblasts. Ectopic expression of miR-9/9^∗^-124 in human adult fibroblasts leads to the switching of several non-neuronal to neuronal components for neural fate acquisition. miRNAs orchestrate the genetic switching of epigenetic regulators including the BAF complex subunits (BAF53a, BAF45a, and SS18 to BAF53b, BAF45b/c, and SS18L1/CREST), TOP2A to TOP2B, and DNMT3B to DNMT3A. These coordinated molecular switches underlie the establishment of a neuronal epigenetic landscape during neuronal reprogramming. Additionally, the switching of PTB proteins, from PTBP1 to PTBP2, mediated by miR-124 activates neuron-specific alternative splicing program.

The BAF complex is crucial for mammalian nervous system as mutations in BAF subunits have been implicated in neurological disorders such as Coffin-Siris syndrome due to mutations in BRG1 and BRM (Tsurusaki et al., 2012; Ronan et al., 2013), and SS18L1/CREST in amyotrophic lateral sclerosis (ALS) (Chesi et al., 2013). During neural development, several BAF complex subunit switches to form the neuron-specific BAF (nBAF) complex (Staahl and Crabtree, 2013). The assembly of the nBAF complex requires the switching of progenitor subunits (BAF53a, BAF45a, and SS18) to neuronal subunits (BAF53b, BAF45b or BAF45c, and SS18L1/CREST) between the proliferating ventricular zone and the post-mitotic zone (Olave, 2002; Lessard et al., 2007; Wu et al., 2007; Yoo et al., 2009; Staahl et al., 2013). These molecular switches also occur during miRNA-mediated direct conversion of human fibroblasts into neurons (Staahl et al., 2013), in which the reciprocal switching of BAF53a to BAF53b is directly orchestrated by miR-9/9^∗^ and miR-124 (Yoo et al., 2009; Staahl et al., 2013; **Figure 1**). The assembly of the nBAF complex is essential for proper neuronal function in learning and memory as loss of function of either BAF53b or SS18L1/CREST dramatically reduced dendritic outgrowth and morphology (Aizawa et al., 2004; Wu et al., 2007; Staahl et al., 2013; Vogel-Ciernia et al., 2013). The function of chromatin remodeling by BAF complex was also found to be critical for neuronal reprogramming as loss of BRG1 during miRNA-mediated reprogramming abolished the chromatin landscape permissive to the activation of the neuronal program (Abernathy et al., 2017).

## Switching of Chromatin Modifiers are Crucial for Cell Fate Conversion

The switching of other homologous epigenetic regulators also occur during neurogenesis and neuronal reprogramming, though may not be direct targets of miR-9/9^∗^-124. These include the switching of DNA topoisomerase II (TOP2) that functions to decatenate and catenate chromatin, from non-neuronal TOP2A to neuronal TOP2B (Watanabe et al., 1994; Tsutsui et al., 2001a; Tiwari et al., 2012; Thakurela et al., 2013). TOP2A is expressed in mitotic cells and interacts with BAF complexes to modulate chromatin accessibility (Tsutsui et al., 2001b; Dykhuizen et al., 2013; Wijdeven et al., 2015; Miller et al., 2017). On the other hand, TOP2B is required for neuronal differentiation *in vitro* and *in vivo* (Yang, 2000; Tsutsui et al., 2001b; Tiwari et al., 2012). Consistent with neuronal differentiation, miR-9/9^∗^-124 instruct the similar switch of TOP2 homologs during neuronal reprogramming of human adult fibroblasts by inducing a rapid reduction of TOP2A in fibroblasts for the selective expression TOP2B in converted neurons (Abernathy et al., 2017; **Figure 1**).

Similarly, the reconfiguration of the epigenetic landscape during neuronal reprogramming also involves changes in DNA methylation patterns (Abernathy et al., 2017). Regarding the reprogramming activities of the miRNAs, it has been shown that ectopic expression of miR-9/9^∗^-124 in fibroblasts recapitulated the molecular switching of *de novo* methyltransferases, DNMT3B to DNMT3A, similarly, to neural differentiation *in vivo* (Feng et al., 2005; Watanabe et al., 2006; Abernathy et al., 2017; **Figure 1**). DNMT3A has been implicated in various aspects of neuronal development, including synaptic plasticity (Feng et al., 2010; Colquitt et al., 2014), but it remains unclear how DNMT3A modulates gene expression in the nervous system. Although DNA methylation is viewed as a repressive mark, methylation marks deposited by DNMT3A have also been associated with enhanced gene expression through antagonizing PRC2 activity (Wu et al., 2010). The dramatic change in DNA methylation profile in miRNA-induced neurons also involves the induction of a family of demethylase, ten–eleven translocation (TET) family proteins (TET1/2/3) (Abernathy et al., 2017), implicated in neuronal development (Hahn et al., 2013; Zhang et al., 2013). It should be noted, however, what additional molecules interact with DNMT3A and TET proteins during neuronal reprogramming to influence DNA methylation at specific loci remains largely unknown.

Although the mechanisms underlying the switching of epigenetic effectors remain to be precisely defined, it is clear that miR-9/9^∗^-124 promote neuronal identity during the direct reprogramming of human fibroblasts through establishing an epigenetic state permissive for the downstream acquisition of neuronal fate. The reciprocal temporal and spatial switching of chromatin modifiers observed both during neurogenesis and neuronal reprogramming highlight the complex and dynamic epigenetic regulations required to overcome cell fate barriers.

## miR-124-Mediated Ptb Switching Regulates Neuronal Splicing Profile

In addition to epigenetic regulators, post-transcriptional regulation of gene expression appears to be integral for neuronal reprogramming. The expression of PTB (polypyrimidine tract-binding) proteins, PTBP1 and PTBP2, are mutually exclusive and exhibit reciprocal switching during neural fate acquisition (Boutz et al., 2007). PTB proteins are RNA-binding proteins that bind to U-rich tracts primarily in introns for the post-transcriptional regulation of mRNAs, including alternative splicing (Wagner and Garcia-Blanco, 2001; Keppetipola et al., 2012). PTBP1 is expressed in non-neuronal cells and neural progenitors whereas the expression of its neuronal homolog, PTBP2 (nPTB), a splicing target of PTBP1, is primarily restricted to post-mitotic neurons in the nervous system (Boutz et al., 2007; Makeyev et al., 2007). PTBP1 represses PTBP2 expression by introducing a premature stop through the skipping of PTBP2 exon (Boutz et al., 2007). During development, the expression of miR-124 at the onset of neurogenesis mediates the switching of PTB proteins by targeting the 3′UTR of PTBP1, thereby alleviating PTBP1-mediated repression of PTBP2 in neurons (Makeyev et al., 2007). Although PTB proteins exhibit functional redundancy (Spellman et al., 2007), PTBP2 in neurons are essential for the proper splicing of various transcripts involved in neuronal function (Boutz et al., 2007; Licatalosi et al., 2012; Zheng et al., 2012; Li et al., 2014). Interestingly, ablating PTBP1 function in several cell types, including mouse embryonic fibroblasts, though insufficient in human fibroblasts, led to the direct conversion into neurons (Xue et al., 2013; Xue Y. et al., 2016), suggesting the significance of PTBP2 for the induction of neuronal fate. In addition to the activation of PTBP2 upon neural fate acquisition, PTBP2 level attenuates later in development for neuronal maturation (Li et al., 2014; Xue Y. et al., 2016). The attenuation of PTBP2 can be recapitulated with sequential knockdown of both PTB proteins resulting in the reprogramming of human fibroblasts into neurons (Xue Y. et al., 2016). The proposed mechanism is that PTBP2 reduction initiates a regulatory loop that activates downstream BRN2 for miR-9 expression, which dampens PTBP2 activity through 3′UTR targeting (Xue Y. et al., 2016). Altogether, PTBP2 level is dynamically regulated throughout neuronal differentiation and is essential as PTBP2 knockout results in neuronal death (Li et al., 2014).

## The Use of miRNA-Induced Neuronal Ground State for Subtype-Specific Neuronal Reprogramming

**FIGURE 2:**
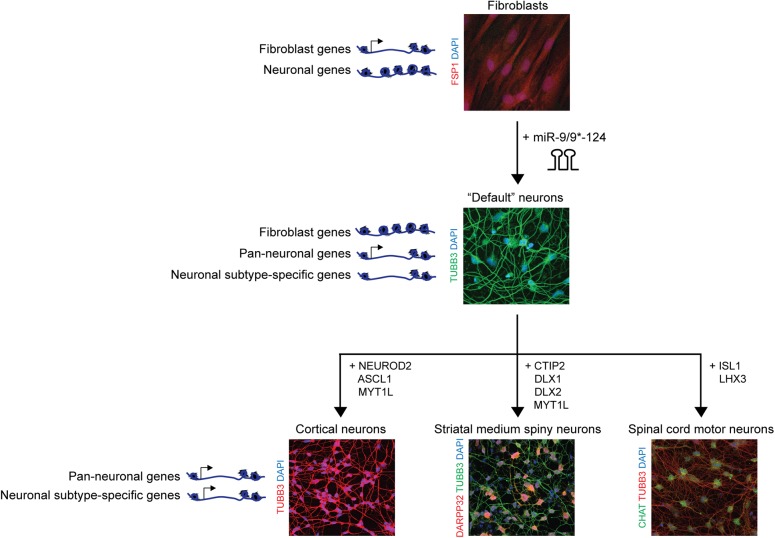
Synergism between microRNAs and transcription factors for subtype-specific neuronal reprogramming of human adult fibroblasts. miR-9/9^∗^-124 mediate the erasure of fibroblast fate through remodeling of a fibroblast-specific chromatin landscape and transcriptome profile and promote a neuronal chromatin landscape and transcriptome. miRNA alone generates a default neuronal state characterized by the reconfiguration of the chromatin state permissive to the subtype-defining inputs of transcription factors. With distinct combinations of transcription factors, the miRNA-based conversion have been successfully employed to generate cortical neurons (Yoo et al., 2011), striatal medium spiny neurons (Victor et al., 2014), and spinal cord motor neurons (Abernathy et al., 2017).

As neurological disorders affect distinct neuronal subtypes, the generation of neuronal subtypes has been of interest not only for dissecting the underlying mechanisms behind subtype-specific neuronal conversion, but also for the implication of the reprogrammed neurons in disease modeling. miR-9/9^∗^-124 have been shown to induce a “default” neuronal state characterized by enhanced accessibility of chromatin regions encompassing neuronal genes (**Figure 2**). These regions include genes specifically expressed in distinct neuronal subtypes, yet remain inactivated, thereby providing the chromatin environment that is open and permissive for subtype-defining inputs of transcription factors (Abernathy et al., 2017). Furthermore, unlike iPSC-based reprogramming methods, direct neuronal conversion bypasses an embryonic intermediate (Lapasset et al., 2011; Miller et al., 2013), thereby retaining the age signatures of starting fibroblasts including the epigenetic clock (Horvath, 2013), age-associated changes in transcriptome and microRNAs, reactive oxygen species (ROS) levels, DNA damage and telomere lengths (Mertens et al., 2015; Huh et al., 2016; Tang et al., 2017). As direct neuronal conversion can faithfully recapitulate age-associated phenotypes, directly reprogrammed neurons hold promise in the modeling of adult-onset neurodegenerative diseases and necessitates the control of subtype-specificity during neuronal reprogramming.

The use of synergism between miRNAs and subtype-defining transcription factors to obtain subtype-specific neurons has been successful in generating cortical neurons (Yoo et al., 2011), striatal medium spiny neurons (MSN) (Victor et al., 2014), and spinal cord motor neurons (Abernathy et al., 2017; **Figure 2**). For instance, heterogeneous population of excitatory and inhibitory neurons belonging to the cortex can be obtained with the use of miR-9/9^∗^-124 in combination with NEUROD2, ASCL1, and MYT1L (DAM) cocktail from adult fibroblasts (Yoo et al., 2011). However, it remains to be tested whether layer-enriched transcription factors would be able to further guide the cortical lineage to neurons with layer-specific identities. Since previous studies demonstrated the plasticity of cortical neurons being able to transition between cortical layer fates (Rouaux and Arlotta, 2010, 2013; De la Rossa et al., 2013), it raises the potential that a similar approach may be taken in a cellular reprogramming context.

An enriched population of striatal MSN, the neuronal subtype primarily degenerated in Huntington’s disease (HD), can be derived using miR-9/9^∗^-124 in conjunction with CTIP2, DLX1/2, and MYT1L (CDM) factors (Victor et al., 2014). More than 70% of cells express DARPP32, a marker of MSNs, and when injected into the stratum of mouse pups, the converted MSNs incorporate and project to the substantia nigra and globus pallidus *in vivo* with electrophysiological properties similar to neighboring endogenous mouse MSNs (Victor et al., 2014). Interestingly, applying the MSN-specific neuronal conversion approach in fibroblast samples from symptomatic patients has proven to be successful in generating patient-specific MSNs manifesting hallmark HD pathology, including HTT aggregation, spontaneous neuronal death, and increased DNA damage (Victor et al., 2018). Importantly, the manifestation of HD-associated phenotypes was dependent on the specificity of the type of neurons generated and the age status in converted neurons, further highlighting the importance of age and subtype-specificity in modeling adult-onset diseases (Victor et al., 2018).

Spinal cord motor neurons are most susceptible to degeneration in amyotrophic lateral sclerosis (ALS) and spinal muscular atrophy (SMA) diseases, and devising a conversion protocol could be instrumental to the study and modeling of motor neuron (MN) diseases. Using a combination of NEUROG1, SOX11, ISL1, and LHX3 (NSIL), MNs can be generated from fibroblasts of ALS patients in which the patient-derived MNs manifest various ALS pathologies, including FUS protein mislocalization and neuronal degeneration (Liu et al., 2016). Alternatively, miRNAs in conjunction with two transcription factors, ISL1 and LHX3, have been shown to generate functional mature MNs that display transcriptional signatures similar to *in vivo* mouse spinal MNs (Abernathy et al., 2017). Despite the robustness in generating a highly enriched population of spinal cord MNs, it remains to be demonstrated whether the MNs derived through the miRNA-induced neuronal state can be used to model ALS or SMA.

The use of transcription factors only, ASCL1, NURR1, and LMX1A, to directly convert fibroblasts of healthy and Parkinson’s disease (PD) patients into dopaminergic cells is possible but with limited efficiency in human cells (Caiazzo et al., 2011). Interestingly, reprogramming efficiency improved with the addition of neuronal miRNA, miR-124, and shRNA against p53, to the transcription factor cocktail using adult fibroblasts (Jiang et al., 2015). One of the proposed mechanisms behind this enhancement is due to the activation of TET proteins, in particular TET1, during reprogramming, as knockdown of TET1 results in increased cell death while overexpression enhances the overall number of TUBB3 and TH positive cells (Jiang et al., 2015). The induction of TET family members has also been observed in miR-9/9^∗^-124-mediated reprogramming (Abernathy et al., 2017).

The generation of additional neuronal subtypes, including serotonergic neurons for the study of neuropsychiatric disorders such as schizophrenia (Vadodaria et al., 2016; Xu et al., 2016) and sensory neurons for the study of pain sensation (Blanchard et al., 2015; Wainger et al., 2015) have been demonstrated using the transcription factor approach. It remains to be tested whether miR-9/9^∗^-124 could be combined with similar transcription factors to enhance overall conversion efficiency in human cells.

## Summary

miR-9/9^∗^-124-mediated direct conversion of human adult fibroblasts into functional neurons reconfigures and establishes a pan-neuronal epigenetic landscape permissive on which brain region-enriched transcription factors can act and generate specific neuronal subtype. miR-9/9^∗^-124 are potent neurogenic molecules as they mediate numerous genetic switches that occur during neurogenesis, in which many include epigenetic players and pro-neurogenic effectors that are important to overcome cell fate barriers and activate neuronal fate programs. As miRNAs regulate expression of multiple genes, the pro-neural environment established by the miRNAs allow for the use of this paradigm for the study of neural fate acquisition. To better understand and address the role of brain-enriched miRNAs, examining miRNA-mRNA network would provide invaluable insights to the acquisition of neuronal fate. Though much remains to be uncovered, with the maintenance of age of starting fibroblasts preserved after cellular conversion, modeling age-dependent neurodegenerative diseases through direct reprogramming allows for the faithful recapitulation of age-associated pathogenesis for mechanistic studies of the disease.

## Author Contributions

Y-LL planned, researched, and wrote the manuscript. AY planned and edited the manuscript.

## Conflict of Interest Statement

The authors declare that the research was conducted in the absence of any commercial or financial relationships that could be construed as a potential conflict of interest.
